# Development of an Unified Food Composition Database for the European Project “Stance4Health”

**DOI:** 10.3390/nu13124206

**Published:** 2021-11-24

**Authors:** Daniel Hinojosa-Nogueira, Sergio Pérez-Burillo, Beatriz Navajas-Porras, Bartolomé Ortiz-Viso, Silvia Pastoriza de la Cueva, Fabio Lauria, Alexandra Fatouros, Kostas N. Priftis, Verónica González-Vigil, José Ángel Rufián-Henares

**Affiliations:** 1 Centro de Investigación Biomédica, Departamento de Nutrición y Bromatología, Instituto de Nutrición y Tecnología de los Alimentos, Universidad de Granada, 18071 Granada, Spain; dhinojosa@ugr.es (D.H.-N.); spburillo@ugr.es (S.P.-B.); beatriznavajas@ugr.es (B.N.-P.); spdelacueva@ugr.es (S.P.d.l.C.); 2 Department of Biochemistry and Molecular Biology, Boonshoft School of Medicine, Wright State University, Dayton, OH 45435, USA; 3 Departamento de Ciencias de la Computación e Inteligencia Artificial, Universidad de Granada, 18071 Granada, Spain; bortiz@ugr.es; 4 Institute of Food Sciences, National Research Council, 83100 Avellino, Italy; fabio.lauria@isa.cnr.it; 5 Department of Food Chemistry and Analysis, Institute of Food Technology and Food Chemistry, Technische Universität Berlin, 13355 Berlin, Germany; a.urbisch@tu-berlin.de; 6 Allergology and Pulmonology Unit, 3rd Paediatric Department, National and Kapodistrian University of Athens, 12462 Athens, Greece; kpriftis@otenet.gr; 7 Gestión de Salud y Nutrición S.L., 33003 Oviedo, Spain; vgonzalez@gsnsoft.es; 8 Instituto de Investigación Biosanitaria ibs.GRANADA, Universidad de Granada, 18071 Granada, Spain

**Keywords:** food composition database, food standardization, food data, nutrients, bioactive compounds, public health, personalized nutrition

## Abstract

The European Commission funded project Stance4Health (S4H) aims to develop a complete personalised nutrition service. In order to succeed, sources of information on nutritional composition and other characteristics of foods need to be as comprehensive as possible. Food composition tables or databases (FCT/FCDB) are the most commonly used tools for this purpose. The aim of this study is to describe the harmonisation efforts carried out to obtain the Stance4Health FCDB. A total of 10 FCT/FCDB were selected from different countries and organizations. Data were classified using FoodEx2 and INFOODS tagnames to harmonise the information. Hazard analysis and critical control points analysis was applied as the quality control method. Data were processed by spreadsheets and MySQL. S4H’s FCDB is composed of 880 elements, including nutrients and bioactive compounds. A total of 2648 unified foods were used to complete the missing values of the national FCDB used. Recipes and dishes were estimated following EuroFIR standards via linked tables. S4H’s FCDB will be part of the smartphone app developed in the framework of the Stance4Health European project, which will be used in different personalized nutrition intervention studies. S4H FCDB has great perspectives, being one of the most complete in terms of number of harmonized foods, nutrients and bioactive compounds included.

## 1. Introduction

There is a close relationship between eating habits, nutrition and health [1]. Many efforts have been made to investigate the nutrient composition of foods consumed by the population [2]. Food composition data describe the content in terms of energy, macronutrients and micronutrients, as well as other compounds such as phytochemicals, antinutrients, bioactive compounds or toxic compounds in foods [3]. Generally, food composition data are published via food composition tables (FCT) and more recently as food composition databases (FCDB) [4,5,6,7].

The FCT/FCDB provide data of foods and beverages consumed by the largest portion of a population [8,9,10]. Currently, there are new agents to take into account, such as climate change [11,12,13] or the loss of biodiversity [12,14]. Add to this the constant change in consumer preferences [15,16], such as the increased consumption of processed products [17], novel foods [12,18], and an increase in global trade [5,19]. Due to these factors, FCDB are increasingly trying to collect a greater number of nutrients, bioactive compounds and foods.

**Figure 1:**
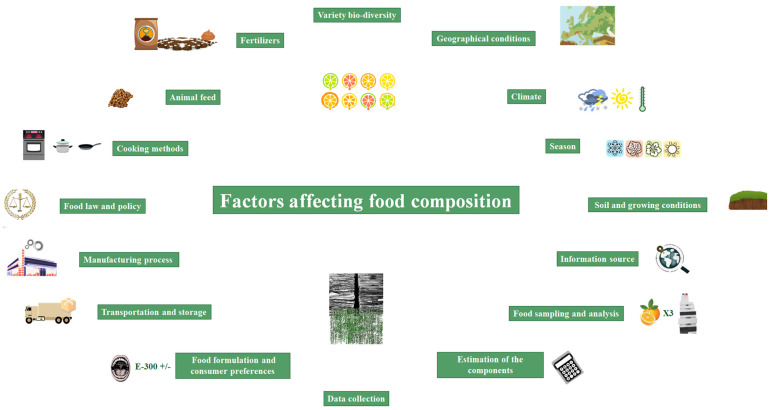
Main factors affecting the nutrient content and food composition. Numbers correspond to appropriate references.

FCDB data can come from: (i) original analytical data; (ii) published or imputed values of a specific or similar food; and (iii) calculated values or data provided by other FCDB [6,9,10]. On the other hand, food composition can be influenced by different factors [5,6,11,15,20,21,22] as depicted in Figure 1. All these factors can result in a somewhat different food composition between countries, and even between regions from the same country, thus requiring the development of more detailed and higher quality FCDB [14,15].

FCT/FCDB are an essential tool in a wide range of areas. For example, in the field of public health [10], health programs and clinical practice [10,23], nutritional epidemiology [24], in research and food safety [18,20,25], in the food industry [20], and in agricultural programs and policies [7,23].

Currently, a growing number of countries are updating their FCT/FCDB, for example, McCance and Widdowson’s food composition table [26], the Dutch Food Composition Database (NEVO) [27] and Frida Food Data [28] which include a wide range of foods and compounds, making them a reference at an international level [12]. However, several countries still lack their own data sets [10,15,20,22,29] so they often resort to foreign FCT/FCDB [9,24] such as the Food and Agriculture Organization (FAO) [30]; or the United States Department of Agriculture (USDA) FCDB [31], among others. Nevertheless, most food composition data are based on fresh foods, while information on processed foods, recipes or fortified foods is usually missing or not up to date [9,15,16,29]. Organizations such as the International Network of Food Data Systems (INFOODS) [23,32,33] are making great efforts to provide information about different FCT/FCDB, promoting the reliability and up-to-date nature of the data [34,35].

Therefore, by having a harmonized and standardized FCT/FCDB, comparisons between countries would be possible and nutritional data would be more accurate and comprehensive [10,15,34]. In order to standardize terms between data bases, different ontologies are used in nutritional research [9,36,37]. The most common is LanguaL™ [38,39]. LanguaL™ is based on the concept that any food (or food product) may be described systematically by a combination of characteristics [39]. There are other descriptors such as those developed by the European Food Safety Authority (EFSA), and FoodEx2 [40]. FoodEx2 is a standardized food classification that consists of individual food items aggregated into food groups and food categories in a hierarchical structure [15,37,40,41,42]. Recently, efforts have been made to map FoodEx2 facet descriptors with LanguaL codes [39,42].

In order to overcome this challenge, in the last decades great progress has been made to develop standards and guidelines focused on the harmonization and standardization of FCDB [10,15,23]. Among the most important is FAO/INFOODS that coordinates food composition activities at the international level [6,32]. FAO/INFOODS has developed different strategies in an attempt to harmonize data and make it comparable across countries [4,6,32,33,43,44,45,46].

Additionally, there have also been numerous EU-funded initiatives to standardize and harmonize food compositional data [15] such EUROFOODS, COST99 or NORFOODS [16]. More recently, they continued via the European Food Information Resource Network (EuroFIR), now known as EuroFIR AISBL [2,10,16,47]. The main objective of EuroFIR is to contribute to the harmonization of high-quality food composition data in Europe [47,48,49]. For this purpose, the EuroFIR project has developed different tools like its own LanguaL™ descriptors; EuroFIR Theasauri or FoodEXplorer. FoodEXplorer is a query tool that includes food composition data across more than 30 countries [20,47] and is updated regularly [2,47,50]. In Europe, these networks allowed the development of large multicenter nutritional studies. For example, the European Prospective Investigation into Cancer and Nutrition (EPIC) [51]. Notably, food composition analysis is very expensive and can be time consuming [22,46]. However, an increasing number of FCDB are introducing as many nutrients and bioactive compounds available as possible [30,52,53,54]. FooDB (https://www.foodb.ca, accessed on 27 October 2021) represents the most comprehensive effort to integrate food composition data [24] and a large amount of different compounds [55].

Stance4Health (Smart Technologies for personalized Nutrition and Consumer Engagement) (S4H) is a project funded by European Union’s Horizon 2020 research and innovation program, aimed at evaluating the benefits of a novel smart personalized nutrition service in a large clinical study [56]. One of the main tasks of the project is to build a nutritional database (with as many foods and nutrients as possible) to complete the national FCDBs from the countries involved in the project. As the FCT/FCDB of the countries is completed, a more accurate approximation of the users’ diets will be achieved.

The aim of the present study is to describe all the harmonization efforts and introduce this novel and unified Stance4Health’s FCDB (S4H FCDB). This database will be part of the app developed in the framework of the European project, which will be used in different personalized nutritional intervention studies (Trial ID: ISRCTN63745549).

## 2. Materials and Methods

### 2.1. Working Group Organization and Training

A working team composed of two coordinators and a committee (including researchers, computer scientists and compilers, all of whom were dietitians and nutritionists) was established for the preparation of the S4H FCDB. Both the coordinators and the compilers completed the e-learning course offered free of charge by FAO/INFOODS [57]. The e-FoodComp course on food composition was designed by experts to be used by different professional users. The course consisted of 14 lessons structured in five units, for a total of approximately 10 h. The course offers a large number of examples and exercises suitable for on-the-job training. In addition, different guides and research were chosen to be used as a reference for the standardization and harmonization processes [3,25,32,33,41,42,43,44,45,46,47,48,49,50,51,57,58,59,60,61,62,63,64,65,66,67,68]. The coordinators established the general guidelines, and also helped choosing and obtaining the FCDBs used. In addition, they were subsequently responsible for checking and assessing the quality of the harmonized procedures and data. The remaining committee members performed the rest of the tasks.

### 2.2. Data Collection, Harmonization and Standardization Methods

A personalized nutrition intervention for different populations in Spain, Germany and Greece will be carried out within the S4H Project [56]. For this reason, the three national FCT/FCDB of the intervention countries were used as references [69,70,71]. These FCT/FCDB were completed with values of nutrients, bioactive compounds, such as polyphenols, and foods from different databases [14,26,27,30,31,72,73,74,75] (Table 1). All FCT/FCDB were either free of charge or permissions were granted when needed. The original FCDB data, such as original name or food identifier, were kept for the purpose of future checks or updates. In addition, quality and traceability of the documented data was guaranteed. However, the data needed to undergo some conversions before being added to our FCDB. All data were harmonized in order to obtain standardized foods and nutrients. Subsequently, all the information was entered into dynamic spread-sheets that related the data and characteristics to each other. As all foods were not in one single language, names and recipes were translated into English. All foods were uniquely identified using the standardized food classification and description system proposed by EFSA FoodEx2 [40,42]. The coding was carried out by qualified compilers and the last version of FoodEx2 system was used [40]. FoodEx2 allowed coding of all foods and beverages present in the FCDB into 20 main food categories, divided into subgroups up to a maximum of four levels [68]. Fortified foods, dietary supplements, food commercial brands, recipes or prepared dishes were discarded from the FCDB. Cooked foods were included, and the cooking method was extracted as an additional data element. Generic unbranded processed foods (such as canned foods, pickles, processed meats or pastries, among others) were also included.

The complete dataset was examined and converted into standard units [3,43]. The tagnames for food components developed by INFOODS were used for this purpose [33,60]. In order to ensure harmonization, standard tagnames were designed for each compound. The original FCDB compounds that were in different units or did not correspond to those described in the INFOODS tagnames, were transformed and recalculated to match the one expressed in the standard tagname (i.e., change of units from grams to milligrams) [33,43]. Only in specific cases were tagnames not modified (as in the case of some polyphenols) where the coordinators decided that it was more functional to leave all compounds with the same units. Those compounds that did not have labels were assigned one that was proposed by compilers. The labels and units can be found in Supplementary Material S1 (Excel sheet). All compounds were expressed in amount per 100 g or 100 mL of food and edible portion values were extracted for further calculations as recommended [3,33]. All changes were made manually or semi-automatically in spreadsheets. All changes were monitored and subsequently validated as described in Section 2.5.

### 2.3. Mapping and Unification Process

Once the data were harmonized, a single FCDB was created. The data were differentiated by origin, but organized in a homogeneous structure. The mapping process involved matching foods based on the FoodEx2 identification code. The data were cleaned by eliminating 0 values and treated as missing to eliminate possible errors in the matching. Standard rounding values were taken [43]. Statistical parameters (mean, median, standard deviation) were calculated for each compound whenever a food had the same code. After all the data were evaluated, the coordinators decided to use the median as the final value. Unification was applied to foods with the same codes. The median was used in order to unify and complete the values of a food as long as the matchings were identical. The results were filtered using different filters as values to locate the values of the outer layers. Afterwards, the quality of the data was evaluated. All changes were made in spreadsheets, and Python 3.0 was used for unification and statistical calculations. The scripts used are shown in Supplementary Material S2. For the S4H FCDB, energy was recalculated using the Atwater factors [62]. Once the values were obtained, they could be inputted in the national FCDB for those foods that are not yet included, or for those nutrients or compounds that were missing.

### 2.4. Recipe Calculation and Additional Factors

Recipes or prepared dishes will be introduced as part of another database. Recipes will be linked to the S4H FCDB in order to obtain all the necessary information. For the calculations, the edible portion, cooking method and those factors that can generate changes in the nutrient content (such as retention factors (RF) and yield factors (YF)) will be taken into account. In addition, allergen data and preparation methods will be implemented.

For the harmonized calculation of recipes, a mixed model was used, since it is the most widely used and accepted [3,76]. This method was proposed as standard by EuroFIR, and consist of applying YF at the recipe level and the RF to each individual ingredient [48,77]. This procedure requires incorporating beforehand the standardized YF and RF based on the food group classification system [25,78]. YF and RF values were obtained from different sources in order to cover the largest number of foods and cooking methods [26,50,76,77,78,79,80]. For the RF of polyphenols, in addition to those given in Phenol-Explorer 3.6 [75], the values retrieved from the EPIC study [61] were also used. The calculation method involved the following steps: first, weights of the raw ingredients were collected. Second, nutrient and compound levels were corrected for edible portions, if applicable. Next, ingredients were modified to account for the effects of cooking by using yield factors to adjust the raw weights. In addition, retention factors were also applied for nutrient losses or gains during cooking. Finally, the ingredient values were summed to obtain recipe values. Final values were expressed per 100 g of recipe and per total recipe weight. The estimates were performed automatically and entered as recipes in the database.

### 2.5. Information Management and Data Quality

Tables and FCDB were implemented in MySQL open-source software. MySQL is a cross-platform relational database management system. A total of eight tables were implemented and interrelated. Tables were disaggregated to provide more versatility and security. All values were subjected to a variation range. Organizations such as INFOODS or EUROFIR propose different methodologies to ensure and validate data quality [25,33,50]. However, in this case, the coordinators decided to follow a system of hazard analysis and critical control points (HACCP) [50]. For each data input, an original document and a working document identified with the same code were stored. For each step identified as HACCP, a series of validation tests were performed. These tests were based on different recommendations [3,25,33,50,57]. The validation procedure was followed by corrections, if necessary. The corrections of the conflicting foods were checked by data traceability extending to the original FCDB. The verifications performed are shown in Table 2. Those processes were applied at each stage of quality control, trying to minimize systematic and random errors. All tests were performed manually or semi-automatically by the coordinators, except for the recipes, which were automated.

## 3. Results

Around 26,200 foods were collected from different FCDBs. Branded foods, recipes or ready-to-eat products, among others, were excluded and a total of 6410 foods were obtained. The Netherlands, the Italian and the United Kingdom’s FCDB were the ones that contributed the largest number of foods in the unification process. A large number of foods were excluded from the FAO FCDB due to incomplete information. Subsequent to unification, filtering and quality validation, 2648 foods were obtained for the S4H FCDB and 47% of them had an equivalent food in another FCDB, so that achieved unified values. The foods were grouped by food groups and shown in Supplementary Material S1 (Excel sheet).

Regarding nutrients, bioactive compounds and other information, 880 items were collected. About 95% of the items corresponded to nutrients or other food compounds. Only 5% corresponded to other items such as the food group, its code or some additional factors. During harmonization and standardization, 78.7% of the tagnames were kept with the recommended INFOOD standards units [33,60], without taking into account the polyphenol tagnames. However, the majority of the polyphenols did not have standard tagnames and represented 55.7% of the total of items. Only 5.3% of other compounds did not have standard tagnames. The standard units of 8.4% of the total number of compounds was modified to more functional units.

**Figure 2:**
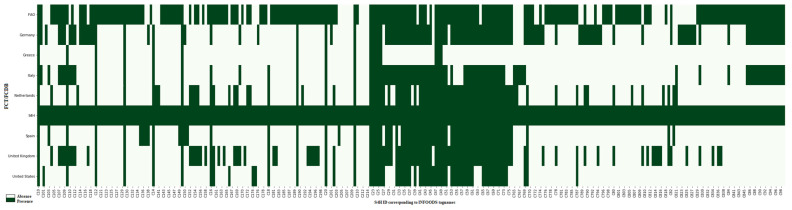
Absence or presence of different compounds and nutrients in the FCDB. The FAO/INFOODS tagnames are expressed with the S4H IDs, listed in the Supplementary Material Table S1. Not all tagnames are shown; the Phenol-Explorer Database is not included and the complete figure is depicted as Supplementary Material Table S3.

Germany contributed the highest percentage (15%) of total nutrients, Spain 9% and Greece 2%. It should be noted that 65.5% of the nutrients included in the database were polyphenols from Phenol-Explorer 3.6. If we do not take this into account, the percentages are tripled, as shown in Figure 2. For example, Spain and Germany had around 88% of the 40 most used nutrients in epidemiology, while Greece had only 40%. After Phenol-Explorer, the FAO FCDB is the one with the highest percentage of compounds, around 28.2%. However, the English and Italian FCDBs were the ones with the highest percentage of nutrient values used in epidemiology, with more than 95%.

**Figure 3:**
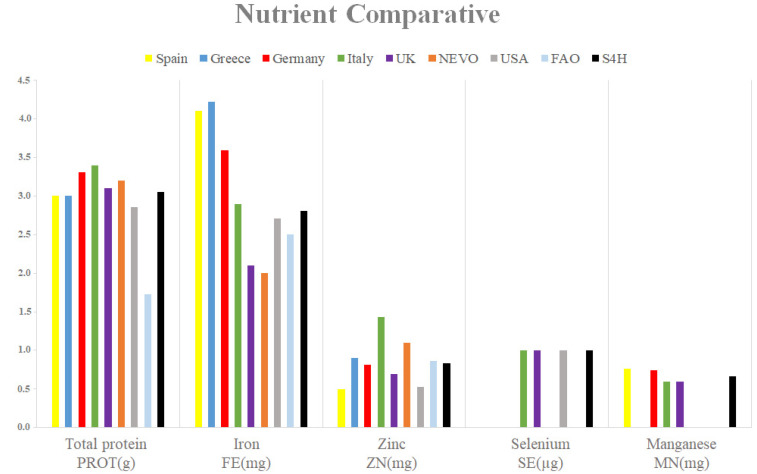
Protein, zinc, selenium and manganese content of the food categorized as spinach in different FCDBs and the unified values corresponding to the S4H FCDB.

Figure 3 shows an example of the values of the unification process for the item A00MH Spinaches, raw. Raw spinach was selected because it was included in most FCT/FCDB. The value of total proteins is quite similar, which confirms a correct classification of the food. However, micronutrient values were more heterogeneous among the different FCT/FCDB. With the unification, the S4H FCDB obtained intermediate values considering the possible variability and also, in the case of Selenium, it retrieved values similar to those of the national FCT/FCDB.

Regarding recipes, tables and interrelations for energy, protein, carbohydrate, fat, sodium, calcium, riboflavin, Vitamin C, the flavonols group and (-)-epicatechin were checked for correctness. A set of recipes was selected from the database to perform manual and automatic calculations; the results were identical in 80% of the cases, and when they were not, mismatches came from the compilers’ failure to choose performance or retention factors. This problem disappears when automated.

After data validation, no errors were detected in the transformation of units because there were no systemic deviations detected in any specific nutrient or compound. In 1.8% of the foods, some nutrients showed extreme standard deviations, most likely coming from the original FCDB. In addition, 7.5% of the foods had high deviations in some nutrients; all of these values, coming from the harmonization and coding phase, were reviewed and corrected. No differences were detected when using either mean or median values, except in some specific cases, such as unified foods with more than six FCDB. Nevertheless, the median value gave estimates closer to the overall computation of the data. In addition, 4.9% of foods had macronutrients that did not meet the established quality limits; the same happened with the sum of total fats, where 2.8% presented mismatches. Therefore, 17% of the food products had some type of error. Of this percentage, about 88% could be resolved by excluding 54 food items, resulting in a total of 2648 foods. The data were transferred properly and all MySQL interrelations were checked.

## 4. Discussion

The aim of this study was to develop a FCDB as complete as possible in terms of food, nutrients and other compounds. This is especially important because globally there is a large nutritional data gap [33]. This trend is changing, since according to Finglas et al. [49] many countries are making efforts to create or update their FCDB. Epidemiological studies where several countries are involved, such as the EPIC study, are becoming more and more common. According to Slimani et al. [51] during the EPIC study a total of 26 nutrients for more than 550 foods per country were selected; after appropriate standardization they were used for cross-country comparisons.

Since the S4H project involves several countries, we used the EPIC study as our reference [51]. All the databases were chosen by agreement between coordinators and researchers. The three countries involved in the intervention were used as the main sources for FCT/FCDB [69,70,71]. The national FCT/FCDB were selected assuming that the most reliable values were available at the local level. Some nutrients were missing from one or more FCDBs. Therefore, we decided to include three more to make ours more representative of European foods [10]. These widely recognized databases were from Italy [72], the Netherlands [26,27] and the United Kingdom, since these countries had more updated versions [81,82]. Finally, four more international FCT/FCDB were included to enrich nutritional composition: the USDA FCDB, since it is widely used [31], the INFOODS/FAO—FCT/FCDB [14,30] to increase the number of nutrients and to take into account the biodiversity of some foods, and, finally, Phenol explorer 3.6 was chosen [73,74,75] due to the great implication that polyphenols have on diet and health [61]; this allowed for the enhancement of national FCT/CBDT through the addition of more foods and the inclusion of more than 600 bioactive compounds.. We discarded 75% of the foods since quality issues were reported in the estimates when introducing new commercial foods [83], emerging dietary components [61], fortified foods or dietary supplements [84], since these are specific to each country. Recipes and prepared dishes were also not incorporated due to the great variability of preparations in each country [85]. Recipes will be linked from another interconnected database under construction. The national FCDB will input those foods or missing values from the S4H FCDB.

### 4.1. Standardization and Unification

The use of food composition data from different countries needs a high level of harmonization of both food values and the nutrients that are included [48]. Data processing requires precise nomenclature and standardized methods, such as the use of ontologies or tags that allow correct classification and description [86]. Nutrients from the TEDDY study were compared between four countries. According to Uusitalo et al. [67], harmonizing datasets before calculations generally made the results comparable, as systematic and random errors were minimized. This approach was previously used for ten European countries in the EPIC study, producing similar results [51,66].

Due to the large amount of available food items, the implementation of artificial intelligence and computational approaches is recommended [87]. Currently there are many automatic and semi-automatic tools that are extensively used to classify FCDBs [9,41,87,88]. A clear example is the ASA24 system that uses automated methods for several databases [16,87]. Another example is StandFood, a semi-automated system that obtained an overall result accuracy of 79% [41]. New techniques of natural language processing [88], machine learning, and statistical models, such as Monte Carlo simulations [12] or extraction of ‘big data’ [20], make the process faster than manual work [16]. However, due to the complex work, a manual *post hoc* review is always required [82,87]. After a first approach using different methodologies, manual and semi-automated harmonization and standardization work was decided to be performed in the S4H FDCB. Although human errors are still possible, this work guarantees a higher accuracy when comparing the same foods from different FCDB than automated predictions [41].

The first step was to achieve harmonization to classify foods. Durazzo et al. [37] classified foods based on different criteria. One of the classifications used is the FoodEx2 classification implemented by EFSA [40]. We selected these classification criteria due to its hierarchical nature and its widespread use. All foods were harmonized and linked between the different FCDBs. This classification provides the possibility to match foods, although full comparability is not guaranteed [2,59]. Secondly, since all nutrients and compounds have to be made comparable, they were defined in the same way, according to measurement units [51,67]. The tagnames proposed by INFOODS [43], indicating the name of the component, units and analytical method [60], have been implemented in different FCDBs around the world [45]. INFOODS tagnames allowed us to normalize variables from all databases to reference units (such as μg or mg) with faster results. Also, when unifying two nutrients, it allowed us to ensure that they were expressed in the same way and could be comparable. We modified 8.4% of the units of the tagnames to obtain a more functional FCDB. Most of the individual phenolic compounds did not have a tagname, and a new tagname was created to facilitate their integration into S4H FCDB. After standardizing both foods and nutrients, we had the opportunity to unify those foods that were categorized as identical. This would allow the inputting of those missing data and foods in the national FCDB.

Several studies claim that for research purposes in nutritional epidemiology, it is better to approximate nutrient values than to leave them as missing [51]. Not imputing data could lead to systematic underestimations of nutrient intake [18]. Although authors and institutions recognize this as a reliable method [33], others are critical, arguing that food composition changes considerably from one country to another [2]. S4H FCDB inputted the values of a weighted estimate of several FCDBs, making the values of high quality and taking into account the biodiversity of foods, thereby improving the estimations [14]. The inputting of missing values are frequent mechanisms that are performed when using FCDBs with recognized data quality [88]; typically, the data come from FCDBs from the United States, Europe, or other countries in the same region [5,12]. An example is the FCDBs from countries in sub-Saharan Africa, which import up to 88% of data about animal-source food [22]. Another example is the Middle East FCDBs, which inputted food from the United Kingdom FCDB [81]. The S4H FCDB uses an ad hoc approach to standardize the FCDB, as was done in the EPIC study [66]. This approach will make it possible to add foods or replace the value of a missing compound from other FCDBs with comparable estimated and weighted values [51,66].

During the first unification tests between foods, large standard deviations were identified in some macronutrient or micronutrient, largely coming from beverages and spices. The reason was that most of the 0 values for a compound or nutrient were not of the ‘logical zero’ type. Authors such as Pérez Grana or Westenbrink et al. [1,25] recommend that missing values should never be replaced by 0 and even modify the ‘logical zero’ values so as to avoid affecting the estimations. Before unifying the values, all the 0 values were removed. Then, by unifying the values, most of the data were homogenized, thus improving the results. The loss of the ‘logical zero’ values would not affect the calculations since they should remain at 0 and can be incorporated later.

On the other hand, although the mean and median were calculated, median values were chosen as the reference value after unification. Although some authors choose the mean [9,25], the median value is, in some cases, a better measure of central tendency [88], especially for extreme values from national FCDB. This ensures homogenization of the data and prevents wrong estimations. The unification allowed the inclusion of many foods and compounds. Figure 3 shows how the unification guarantees the homogenization of values. Once the values were unified and cleaned, as recommended by FAO or EuroFIR [2,33], estimated energy was recalculated using the Atwater coefficients [62].

Organizations such as FAO work with spreadsheets due to their simplicity, wide availability and familiarity to users [44,89]. Our work started out using spreadsheets, although the amount of data quickly became rather difficult to handle [89]. Therefore, the software MySQL was used, which allowed us to send and retrieve data through its interrelated tables [45,90]. This ensured traceability and quality controls, and also facilitated the relationship of S4H FCDB with the recipe tables for subsequent calculations.

### 4.2. Data Quality and Recipe Calculation

High quality data are essential for nutritional studies [48]. The use of the HACCP system [50] allowed us to quickly and sensibly evaluate data quality at different stages. In addition, the FAO guidelines served as a reference in the detection of critical points at any stage of the process [33]. Initial training was essential to successfully complete all the tasks, while guaranteeing the highest possible accuracy and quality.

For S4H FCDB, name verification and food description, as well as translations, were corrected thanks to the collaboration of researchers whose first language was mostly the language of the FCDB.

An FCDB should be frequently updated. For example, in the TEDDY study, the FCDB was updated at least once a year [67]. The incorporation of the original food IDs to guarantee the traceability of the food was a critical control point. Original food IDs allowed us to identify and correct errors and even to retrieve or update the information. Failures in the classification and verification of food grouping and compound labeling were detected due to outliers or manual coding by using standard deviations. Three different checking approaches were used: (i) Checking that the sum of macronutrients was within the range or the presence of implausible values detected semi-automatically in the spreadsheets; (ii) Checking for data transfer from spreadsheet to MySQL by direct verifications between versions and table relationships; and (iii) Checking the model recipe by manual verifications by compilers and automatic verifications by interconnecting the different databases. These verifications made it possible to ensure the comparability and reliability of the data.

Performing chemical analyses for all recipes and complex food matrices is not achievable. Calculations are performed indirectly using each ingredient’s nutritional information [10,91]. In order to properly calculate a recipe, different parameters must be taken into account, such RF or YF. One of them is that values should not be missing, since these may lead to a biased underestimation of nutrient intake [43]. During unification and the inputting of values, this problem was solved to a large extent. The EuroFIR recipe calculation procedure was selected as a reference because it is one of the most commonly used [76]. There are several studies that use an app or software to estimate or perform interventions in nutrition and health [61,92,93]. Accordingly, the S4H FCDB will be interlinked with a recipe database. It will therefore make possible the automatic calculation of recipe values, taking into account all necessary parameters, such as edible portion, retention factors and yield factors or even allergens. Thus, the recipes will be as adequate and representative as possible to cover the needs of the population.

### 4.3. Strengths and Limitations of the S4H FCDB

With the continuous expansion of food trade worldwide [10], climate change or innovation in agriculture [13], international FCDBs are essential. For this reason, S4H FCDB wants to be a reference in the creation of a unified FCDB. Much effort has been made to overcome the common drawbacks that are generally associated with the FCDB’s construction. The variability in food composition (when using different FCDBs) is one of the most detected limitations [7,20]. S4H FCDB attempts to address this limitation by using the median value as the reference estimation. Additionally, there is no guarantee that national FCDB data are free of errors [2]. However, all national FCDBs are used in their own country. The unification gave us a global view of possible wrong values, allowing them to be corrected. Another limitation was represented by missing foods and nutrients from the national FCDB [47]. The S4H FCDB inputs those missing foods and compounds giving coverage and completing those values in the national FCDB. Discrepancies may exist between the tagnames proposed by FAO/INFOODS and their units [15]. However, the decisions to change units were consensual and made to improve their functionality. Moreover, inputted values from other datasets, especially dishes and recipes, did not guarantee directly related values [10,65]; for this reason, recipes and ready-to-eat products were removed. Recipes will be calculated thanks to the interconnection between the S4H FCDB and a recipe database.

The work was complex, and although the compilers were experts in nutrition, mistakes may have been made when choosing codes for harmonization [15,23]. However, the use of guidelines and data validation throughout the whole process allowed for the verification and correction of possible mistakes. The preparation of this material required a long time, and perhaps with automated methods and a subsequent exhaustive check, similar results could have been obtained [88]. There may have been failures during the translation of some foods [47], especially regional foods, although if no reliable translation was found, foods were discarded. Even so, our results are encouraging. Misspellings and translation mistakes were detected while manually identifying and classifying. Thus, one of the limitations may have actually been a strength.

In most nutritional epidemiological studies, results are similarly interpreted regardless of how they make estimations or which FCDB is used. This generates an unrealistic relationship of nutrient intakes and their impact on health [94]. An increasingly large number of epidemiological studies attempt to make their data comparable [51,67,95,96]. One of the strengths of the S4H FCDB is that with unified values, data from different countries could be compared, as it would take biodiversity and different parameters affecting the same kind of food into account. Another option is to use national FCDB data and only fill in the missing nutrients and compounds to avoid underestimations [6,18]. Organizations such as EUROFIR have the potential to create a standardized FCDB which should be free to use [48]. EFSA already has a tool as a first step towards the unification of nutrients [97]. The S4H FCDB is one of the most comprehensive FCDB regarding the number of foods and nutrients, being able to collect more than 800 compounds from each foodstuff. Thus, to date it is only surpassed by the https://foodb.ca (accessed on 27 October 2021) project supported by the Canadian Institutes of Health Research and by The Metabolomics Innovation Centre. This Database includes not only nutritional information, but also a large amount of bioactive compounds [24,55,98]. However, it must be noted that the S4H FCDB uses different FCT/FCDB, giving much more homogeneous and comparable nutritional values.

### 4.4. S4H FCDB’s Future Perspective

**Figure 4:**
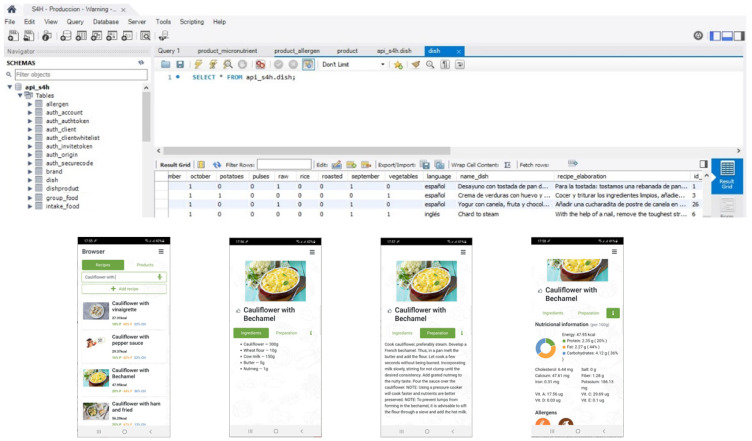
Shows the relationship between MySQL tables and how the recipe is generated in the APP.

The S4H FCDB consists of interlinked tables that make a complete nutritional information system. S4H FCDB not only allows accurate calculations, but also provides the user with information on different aspects integrated in the personalized nutrition system. The purpose of the S4H FCDB was for it to be used in epidemiological studies, in particular precision nutritional studies. This S4H FCDB will be connected to an app that will be used during the nutritional intervention of the project. The study aims to generate personalized nutritional recommendations to different populations, more specifically adults and children [56]. The app derived from the S4H Project will be an automated diet evaluator and generator used from smartphones. A set of more than 10,000 recipes from all countries is expected to be available. All recipes will be implemented in a mobile app for future nutritional intervention. An example is depicted in Figure 4. Other similar apps have a smaller number of foods and were developed from a smaller number of food data sources [82].

The S4H FCDB will be able to connect to other tools. One of the milestones of personalized nutrition is to understand the health level of the gut microbiota of a given patient. The S4H FCDB generated data will also be completed with the use of AGREDA [99], an extended reconstruction of diet metabolism by the human gut microbiota. The S4H FCDB will also introduce commercial products, incorporating allergens and different scores as used in Open Food Facts [16]. These products and fast foods from the different countries of the project will make S4H FCDB more comprehensive and representative [38]. The data will also be updated periodically to avoid obsolescence [5], which will be possible thanks to traceability.

In the future it is expected that the S4H FCDB will be extended by implementing toxic substances, such as food processing contaminants, as few FCDBs contain these components [15,88,100,101,102]. Due to the importance of climate change in nutrition, sustainability parameters and different markers of climate change would be an added value to be included [13]. Finally, in order to identify food-disease associations [55], food biomarkers could be introduced by linking them to FOBI (Food-Biomarker Ontology) [36], or extending the compounds related to https://foodb.ca (accessed on 27 October 2021) or other big data sources [24,98].

## 5. Conclusions

S4H FCDB was built through a huge scientific work to collect and harmonize all the nutritional data. S4H FCDB is one of the most comprehensive FCDB with more than ten FCDBs used, which is one of its main unique characteristics. This food database is comparable to that used in other relevant studies, such as EPIC. A large number of harmonized foods (over 2000) and more than 800 nutrients and bioactive compounds (such as polyphenols) have been included, the inclusion of such a large number of bioactive compounds being another unique strength of the paper. S4H FCDB attempts to mitigate the usual limitations, such as variability in food composition, errors, and missing values in the national FCT/FCDB databases. Trained personnel following the guidelines of official agencies were able to homogenize the information. This made it possible to unify foods, their nutrients and bioactive compounds among the FCT/FCDBs using the median value as the reference value. The values obtained were less extreme and made it possible to complete the national FCT/FCDB. The S4H FCDB has many perspectives, not only the implementation in nutritional studies through an application. But it is also capable of being part of other tools and has the versatility to be continuously enhanced with much more information. Thus, S4H FCDB becomes a solid and indispensable tool to approach the age of personalized nutrition.

## Figures and Tables

**Table 1:** Compilation of FCDBs used in the construction of the S4H FCDB.

Name of the FCDB	Last Update	Nº FoodsT ^1^	Nº Foods ^2^	Nº Items ^3^	References
Tabla de Composición de Alimentos de Martin Peña actualizada de la version original por i-Diet (Spain)	2019	726	711	90	[69]
Composition tables of foods and Greek dishes (Greece)	2007	88	84	18	[71]
Bundeslebensmittelschlüssel (BLS) (Germany)	2014	936	715	146	[70]
Banca Dati di Composizione degli Alimenti per Studi Epidemiologici in Italia (Italy)	2015	978	976	97	[72]
Dutch Food Composition Database (NEVO) (Netherlands)	2019	2152	949	144	[27]
McCance and Widdowson’s ‘composition of foods (United Kingdom)	2019	2910	1208	280	[26]
Food and Nutrient Database for Dietary Studies (FNDDS) (United States)	2018	7083	609	69	[31]
FAO/INFOODS Analytical food composition database version 2.0 (AnFooD2. 0)	2017	2953	346	378	[30]
FAO/INFOODS food composition database for biodiversity (BioFoodComp4.0)	2017	7953	355	538	[14]
Phenol-Explorer 3.6 database on polyphenol content in food	2015	458	457	520	[73,74,75]

^1^ Number of foods used out of total. ^2^ Number of foods included. ^3^ Number of items collected, including information on food nutrients and other compounds and data.

**Table 2:** Steps identified as HACCP and validation testing.

Validation Testing	Step as HACCP
Verification of food name and description, possible misspellings or translation errors.	Harmonization
Classification and consistency verification of food name and grouping.	Harmonization
Verification of FoodEx2 coding and INFOODS compound tagging.	Harmonization
Sum of (water + protein + fat + total carbohydrates + alcohol + ash) is within the range: 95–105 g.	Unification
Implausible values, such total fat value, is = 0, Fatty acids = 0 and Cholesterol = 0, or fiber in fish.	Unification
Outlier values within each nutrient or compound.	Unification
Spreadsheet to MySQL data transfer checking.	Management
Model recipe testing.	Recipes Calculation

## Data Availability

The S4H FCDB is not freely available at the present moment, since it will be used along the research project. Those researchers aiming at using the S4H FCDB should request a license to José A. Rufián-Henares, who will transfer the request to the relevant S4H committee.

## Supplementary Materials

The following are available online at https://www.mdpi.com/article/10.3390/nu13124206/s1, Table S1: INFOODS Tagnames used in S4H FCDB, Code S2: Code used in the S4H FCDB unification stage. Figure S3: Figure with the absence or presence of different compounds and nutrients in the FCDB. All compounds are included and expressed with FAO/INFOODS names.
