# Supplementary figures and images for: Development of an Unified Food Composition Database for the European Project “Stance4Health”
Source: Nutrients. 2021 Nov 24;13(12):4206. doi: 10.3390/nu13124206 (PMC8704708; doi:10.3390/nu13124206)

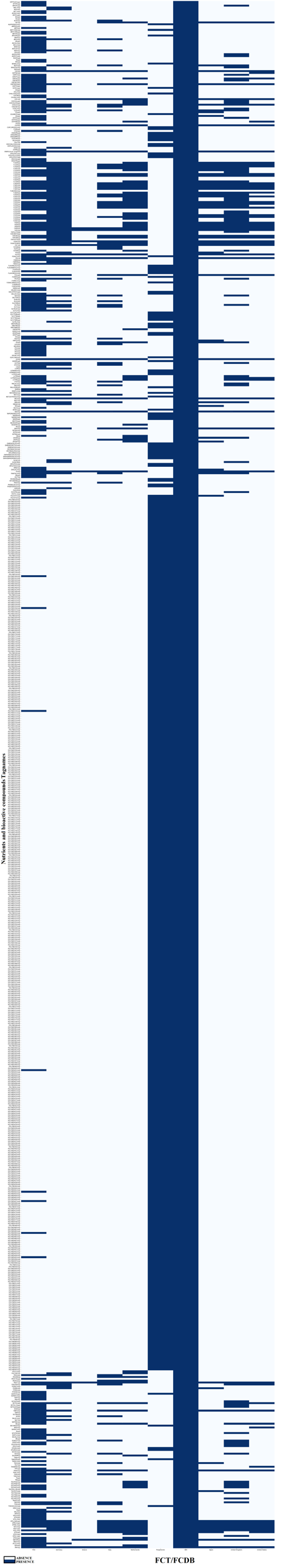

Supplement: Supplementary file 1 [file nutrients-13-04206-s001.zip › Suplemental Figure S3.tif]
